# Reversal of acquired resistance to adriamycin in CHO cells by tamoxifen and 4-hydroxy tamoxifen: role of drug interaction with alpha 1 acid glycoprotein.

**DOI:** 10.1038/bjc.1990.365

**Published:** 1990-11

**Authors:** M. Chatterjee, A. L. Harris

**Affiliations:** Cancer Research Unit, University of Newcastle upon Tyne, Medical School, UK.

## Abstract

Tamoxifen and 4-OH tamoxifen were used to reverse multidrug resistance (MDR) in CHO cells with acquired resistance to adriamycin (CHO-Adrr). Because alpha 1 acid glycoprotein (AAG) can bind a range of calcium channel blockers that also reverse MDR and rises in malignancy, its interactions with tamoxifen and 4-OH tamoxifen were also studied. Tamoxifen decreased the IC50 of 10 microM adriamycin 4.8-fold in the parent CHO-K1 cell line and 16-fold in CHO-Adrr. Similarly 4-OH tamoxifen decreased the IC50 3-fold in the parent cells, but 13-fold in the resistant cells. Tamoxifen and 4-OH tamoxifen were similarly potent in reversing MDR, although their anti-oestrogen potency differs 100-fold. AAG was added in increasing concentrations to the combination of adriamycin and tamoxifen. As AAG concentrations increased from 0.5 to 2 mg ml-1 (the range found in vivo) the effect of tamoxifen on reversing MDR was gradually decreased. At the highest AAG concentrations, there was complete reversal of the effects of both tamoxifen and 4-OH tamoxifen. AAG was found to bind 3H-tamoxifen in a non-saturable non-specific manner, in contrast to the binding of tamoxifen to albumin. Thus the use of tamoxifen as a reversal agent for MDR in vivo may be impaired by high binding to AAG. However, at the lower range of normal values of AAG, there was still an effect of 10 microM tamoxifen. It may be desirable to select patients for modifier studies based on AAG plasma levels.


Br. J. Cancer (1990), 62, 712-717 © Macmillan Press Ltd., 1990

### Reversal of acquired resistance to adriamycin in CHO cells by tamoxifen and 4-hydroxy tamoxifen: role of drug interaction with alpha 1 acid glycoprotein

M. Chatterjee[1] & A.L. Harris[2]

[1]*Cancer Research Unit, University of Newcastle upon Tyne, Medical School, Framlington Place, Newcastle upon Tyne NE2 4HH; and* [2]*ICRF Clinical Oncology Unit, Churchill Hospital, Headington, Oxford OX3 7LJ, UK.*

**Summary** Tamoxifen and 4-OH tamoxifen were used to reverse multidrug resistance (MDR) in CHO cells with acquired resistance to adriamycin (CHO-Adr$^r$). Because alpha 1 acid glycoprotein (AAG) can bind a range of calcium channel blockers that also reverse MDR and rises in malignancy, its interactions with tamoxifen and 4-OH tamoxifen were also studied. Tamoxifen decreased the $IC_{50}$ of 10 μM adriamycin 4.8-fold in the parent CHO-K1 cell line and 16-fold in CHO-Adr$^r$. Similarly 4-OH tamoxifen decreased the $IC_{50}$ 3-fold in the parent cells, but 13-fold in the resistant cells. Tamoxifen and 4-OH tamoxifen were similarly potent in reversing MDR, although their anti-oestrogen potency differs 100-fold. AAG was added in increasing concentrations to the combination of adriamycin and tamoxifen. As AAG concentrations increased from 0.5 to 2 mg ml$^{-1}$ (the range found *in vivo*) the effect of tamoxifen on reversing MDR was gradually decreased. At the highest AAG concentrations, there was complete reversal of the effects of both tamoxifen and 4-OH tamoxifen. AAG was found to bind $^3$H-tamoxifen in a non-saturable non-specific manner, in contrast to the binding of tamoxifen to albumin. Thus the use of tamoxifen as a reversal agent for MDR *in vivo* may be impaired by high binding to AAG. However, at the lower range of normal values of AAG, there was still an effect of 10 μM tamoxifen. It may be desirable to select patients for modifier studies based on AAG plasma levels.

Development of resistance to cytotoxic cancer chemotherapeutic agents is a major impediment to effective treatment of human neoplastic diseases. To study this problem, *in vitro* models of the multidrug resistance (MDR) phenotype have been described where simultaneous cellular-resistance to a number of structurally and functionally unrelated 'natural' anticancer drugs occurred, following exposure to increasing concentrations of a single agent (Biedler *et al.*, 1970). Such multidrug resistance is associated with a decrease in intracellular drug accumulation attributed to a decreased rate of drug influx (Fojo *et al.*, 1985) and/or an enhanced rate of efflux (Dano, 1973; Inaba *et al.*, 1979) or both. The MDR phenotype has been shown to result from increased expression of a gene designated *mdr* (Gros *et al.*, 1986) which is transcribed on to a 4.5-5.0 kb mRNA and the resultant protein product is the 170,000 dalton P-glycoprotein. The concomitant overexpression of P-glycoprotein has been consistently found in different MDR human (Rogan *et al.*, 1984) and animal cell lines (Kartner *et al.*, 1983, 1985).

Modulation of MDR *in vitro* has been demonstrated by several compounds such as verapamil and other calcium channel blockers (Tsuruo *et al.*, 1981), calmodulin inhibitors (Tsuruo *et al.*, 1982), amiodarone (Chauffert *et al.*, 1986) and perhexilene maleate (Ramu *et al.*, 1984*a*). The biochemical basis for some modulators of MDR is their ability to act as a substrate for the active efflux pump mediated by P-glycoprotein, competitively inhibiting the efflux of cytotoxic drugs which bind to P-glycoprotein, and thereby decreasing multidrug resistance (Safa *et al.*, 1987).

A particular problem of using modulators of MDR clinically, is the inability to achieve plasma levels which are effective in reversing MDR *in vitro* without adverse side effects. For example, verapamil is effective in reversing MDR *in vitro* at concentrations 2.2 μM, but maximal effect is seen between 5 and 10 μM. However, maximum achievable levels of verapamil without major side-effects are 5 μM (Benson *et al.*, 1985). In addition, as verapamil binds substantially to alpha 1 acid glycoprotein (AAG), an acute phase plasma protein which increases non-specifically in cancer patients (Paxton *et al.*, 1983), the reversal of MDR by verapamil can be attenuated by the addition of AAG (Chatterjee *et al.*, in preparation).

We were therefore interested in studying compounds where (a) reversal of multidrug resistance was possible, (b) concentrations of drug needed to reverse MDR *in vitro* were achievable *in vivo*, and (c) minimal or no binding to AAG occurred.

We have studied the effect of tamoxifen, an anti oestrogen, on suppression of adriamycin resistance. Ramu *et al.* (1984*b*) have shown that tamoxifen can reverse multidrug resistance in P388/Adr murine leukaemia cells. (Clinically, a daily administration of tamoxifen 20 mg twice daily has been shown by Patterson (1981) to have plasma levels in the range of 450 ng ml$^{-1}$ (1.2 μM) after 12 weeks of treatment.) We have recently observed that high dose administration of tamoxifen (320 mg day$^{-1}$) produced plasma levels of up to 5 μM. Tamoxifen is 99% bound to albumin (Adam, 1981) and no binding with AAG has been reported. Since studies done in cell culture models of MDR are maintained in AAG free medium, we were interested in assessing tamoxifen induced reversal of multidrug resistance in the presence of AAG.

Tamoxifen is metabolised extensively and one of the major metabolites of tamoxifen is 4-hydroxy tamoxifen, which has a 100-fold greater binding affinity for oestrogen receptor and is a more potent anti-oestrogen than tamoxifen (Robertson *et al.*, 1982; Jordan *et al.*, 1977). We therefore wanted to assess the potential of 4-hydroxy tamoxifen as a modulator of adriamycin resistance.

### Materials and methods

### *Drugs*

Adriamycin formulated for clinical use was obtained from Farmitalia, tamoxifen and 4-hydroxy tamoxifen from ICI (UK). Adriamycin was dissolved at 2 mM in water and aliquots stored at −20°C. Tamoxifen and 4-hydroxy tamoxifen were dissolved in 95% ethanol at 10 mM and stored at 4°C. Ethanol at the final concentration present did not affect cell growth. Dilutions of drugs were made in growth medium and

Correspondence: A.L. Harris.
Received 6 March 1990; and in revised form 5 June 1990.

prepared just prior to use. $^3$H-tamoxifen (sp. activity 82 Ci $mmol^{-1}$) was obtained from New England Nuclear. Other chemicals were obtained from the Sigma Chemical Company.

*Cell and culture conditions*

A CHO-Adr$^r$ cell line was isolated from wild-type (CHO-K1) cells by exposure to progressively increasing doses of adriamycin up to a maximum of 0.4 $\mu g\ ml^{-1}$. Both cell lines were maintained in Hams F10 medium (Northumbria Biologicals) supplemented with 5% newborn calf serum, 5% fetal calf serum, antibiotics (streptomycin 100 $\mu g\ ml^{-1}$, penicillin 100 units $ml^{-1}$), nystatin 50 units $ml^{-1}$ and 3 mM glutamine. Cells were maintained as monolayer cultures at 37°C under 5% $CO_2$. The CHO-Adr$^r$ mutant was stable and maintained in drug-free medium. The CHO-Adr$^r$ mutant selected with adriamycin was simultaneously cross-resistant to vinca alkaloids, daunomycin, actinomycin D and colchicine, and has amplification of *mdr* sequences, high *mdr* expression by immunochemistry and high mRNA compared with the parent cell line.

*Quantitation of drug effects*

Drug sensitivity was assessed by a semi-automated colorimetric MTT assay, (Carmichael *et al.*, 1987). Briefly, cells were seeded on 96-well plates. Appropriate drug concentrations were added for 24 h, after which the cells were washed twice with phosphate buffered saline before being placed in 200 µl fresh medium for a further 48 h, 0.1 mg (50 µl of 2 $mg\ ml^{-1}$) MTT was added to each well and incubated at 37°C for 4 h. The medium was then carefully aspirated, crystals solubilised in 100 µl of dimethyl sulphoxide. Absorbences at 540 nm were immediately read on an ELISA multiskan reader. The $IC_{50}$ was defined as the concentration of drug which caused 50% reduction in absorbance. The fold decrease in $IC_{50}$ following addition of tamoxifen or 4-hydroxy tamoxifen was determined by dividing the $IC_{50}$ for the adriamycin treated cells by that of adriamycin plus tamoxifen/4-hydroxy tamoxifen treated cells. A series of controls showed that cell numbers were linearly related to absorbance in the range $5 \times 10^3$ to $4 \times 10^4$, both in the presence and absence of adriamycin and tamoxifen. This absorbence was used in subsequent experiments.

*Tamoxifen binding to AAG or albumin*

To measure binding to tamoxifen to AAG, increasing amounts of unlabelled tamoxifen (0–40 µM) plus $^3$H-labelled tamoxifen (100,000 c.p.m.) were incubated with 1 $mg\ ml^{-1}$ AAG for 1 h at 20°C. The reaction was terminated and unbound tamoxifen removed by addition of 0.4 ml of charcoal-dextran solution (0.5% w/v charcoal; 0.05% w/v dextran) for 15 min at 4°C. The solution was then centrifuged (4,000 r.p.m.) for 5 min at 4°C. Aliquots of 0.5 ml from the supernatant were counted on a scintillation counter. Non-specific binding in the absence of AAG was less than 2%. To assess the binding of tamoxifen in cell-culture medium, binding to 4 $mg\ ml^{-1}$ albumin, which corresponded to 10% serum, was measured as above.

To measure the rate of binding, unlabelled tamoxifen, 10 µM and $^3$H-labelled tamoxifen (100,000 c.p.m.) was incubated with 1 $mg\ ml^{-1}$ AAG for varying time intervals and measured as above.

*Drug accumulation studies*

Exponentially growing cells were harvested by gentle agitation in 0.02% EDTA in PBS, washed by centrifugation and resuspended ($1 \times 10^7$ cells $ml^{-1}$) in PBS, pH 7.4, containing 1% BSA and 10 mM glucose. Aliquots of 100 µl of cells were preincubated for 10 min at 37°C. At time zero, 100 µl of medium containg 1 µM adriamycin, with or without 10 µM tamoxifen, was added and incubated for varying time intervals. Influx was stopped by adding 4 ml of ice-cold buffer (PBS containing 1% BSA), following by centriguation (3,000 r.p.m. × 10 min). The cells were washed twice with ice-cold buffer before the final cell pellet was solubilised in 1% SDS, 10 ml of liquid scintillant was added and radioactivity counted on a scintillation counter.

*Statistics*

Unpaired *t* tests were used to compare data points. Where differences are stated in the text there were significant at $P < 0.05$ or higher degrees of significance. In all figures error bars are shown unless they fall within the size of the symbol.

### Results

*Effect of tamoxifen and 4-hydroxy tamoxifen on CHO-1 and CHO-Adr$^r$ cells*

To examine the sensitivity of the CHO-K1 and CHO-Adr$^r$ cells to tamoxifen and 4-hydroxy tamoxifen, the cells were exposed to appropriate drug concentrations for 24 h (Figure 1a). The $IC_{50}$ of tamoxifen is 21 µM in the CHO-K1 cells and 22.5 µM in the CHO-Adr$^r$ cells. Similarly, the $IC_{50}$ of 4-hydroxy tamoxifen is 26 µM in the CHO-K1 cells and 24 µM in the CHO-Adr$^r$ cells (Figure 1b). Therefore, both cell lines demonstrate similar sensitivity to the parent drug tamoxifen and its metabolite 4-hydroxy tamoxifen. To assess whether the presence of AAG could alter the cytotoxicity of tamoxifen and 4-hydroxy tamoxifen, 2 $mg\ ml^{-1}$ AAG was added. With the addition of 2 $mg\ ml^{-1}$ AAG to increasing concentrations of tamoxifen, the cells were completely protected and cell survival in both cell lines was increased to 80–100% of control. With 4-hydroxy tamoxifen, 2 $mg\ ml^{-1}$ AAG did not cause such a marked effect and the $IC_{50}$ was decreased only 1.4-fold in CHO-K1 cells ($P < 0.05$) and 1.3-fold in the CHO-Adr$^r$ cells ($P < 0.05$). Therefore, the parent compound and its metabolite interact with AAG to different extents.

**Figure 1** Effect of increasing concentrations of (**a**) tamoxifen and (**b**) 4-OH tamoxifen in CHO-K1 (●—●, ○—○) and CHO-Adr$^r$ (▲—▲, △—△) cell lines. Closed symbols, absence of AAG (2 $mg\ ml^{-1}$); open symbols, presence of AAG. Each value represents the mean ± s.e. of at least three experiments.

*Tamoxifen binding to AAG*

To study the binding of tamoxifen to AAG, increasing concentrations of tamoxifen were incubated with either 1 mg $ml^{-1}$ AAG or 4 mg $ml^{-1}$ albumin (corresponding to 10% serum). Increased binding of tamoxifen to 1 mg $ml^{-1}$ AAG occurred with increasing concentrations of tamoxifen (Figure 2). At the highest concentration of 40 μM, 30 nmol $ml^{-1}$ are bound to AAG. The binding is non-specific and non-saturable. It occurs rapidly and is temperature-independent (Figure 3). In contrast, with 4 mg $ml^{-1}$ albumin maximum binding of tamoxifen was 7.5 nmol $ml^{-1}$ and was saturable (Figure 2).

*Effect of tamoxifen on adriamycin cytotoxicity*

Increasing concentrations of tamoxifen (1, 5 and 10 μM ) that had little or no effect on cell growth enhanced the ability of adriamycin to inhibit cell growth in both cell lines (Figure 4a). The $IC_{50}$ of adriamycin in CHO-K1 cells in the absence of tamoxifen was 0.24 μM. With the addition of 1, 5 and 10 μM tamoxifen, the $IC_{50}$ was decreased and the corresponding decrease was 1.8-fold, 2.4-fold and 4.8-fold respectively. In the CHO-Adr$^r$ cells, the $IC_{50}$ of adriamycin was 6.4 μM, and a 26-fold resistance to adriamycin was present. The addition of 1, 5 and 10 μM tamoxifen increased the chemosensitivity of adriamycin and the decrease in the $IC_{50}$ was 2.1, 4.0 and 16-fold respectively (Figure 4b). The shift in the aborbence curves to the left with increasing tamoxifen concentrations was greater in the CHO-Adr$^r$ cells than in the CHO-K1 cells. At the highest concentration of 10 μM tamoxifen, a residual 2-fold resistance in the $IC_{50}$ of adriamycin remained in the CHO-Adr$^r$ cell line, compared to the $IC_{50}$ of adriamycin alone (0.2 μM) in the CHO-K1 cell line.

**Figure 2** Measurement of binding of tamoxifen (0–40 μM) to 1 mg $ml^{-1}$ AAG (●—●) or 4 mg $ml^{-1}$ albumin (○—○). Each point represents the mean ± s.e. of at least three experiments.

**Figure 3** Measurement of rate of binding of 10 μM tamoxifen at 0°C (○—○), 20°C (△—△) and 37°C (□—□) to 1 mg $ml^{-1}$ AAG. Each point represents the mean ± s.e. of at least three experiments.

**Figure 4** **a**, Sensitivity of CHO-K1 cells to adriamycin in the absence (●—●) and presence of 1 μM (○—○), 5 μM (□—□) and 10 μM (■—■) tamoxifen. Each point represents the mean ± s.e. of at least three experiments. **b**, Sensitivity of CHO-Adr$^r$ cells to adriamycin in the absence (▲—▲) and presence of 1 μM (△—△), 5 μM (□—□) and 10 μM (■—■) tamoxifen. Each point represents the mean ± s.e. of at least three experiments.

*Effect of 4-hydroxy tamoxifen on adriamycin cytotoxicity*

To determine the effect of non-toxic concentrations of 4-hydroxy tamoxifen on adriamycin cytotoxicity, 5 μM or 10 μM 4-hydroxy tamoxifen was added to increasing adriamycin concentrations. In both CHO-K1 and CHO-Adr$^r$ cell lines, the addition of 4-hydroxy tamoxifen shifted the absorbance curve to the left, the shift being greater in the CHO-Adr$^r$ cells. In CHO-K1 cells, the $IC_{50}$ of adriamycin was decreased 2.4 and 3.0-fold with 5 μM and 10 μM 4-hydroxy tamoxifen respectively (Figure 5a), whereas in the CHO-Adr$^r$ cells the $IC_{50}$ of adriamycin was decreased 2.0 and 13-fold with a similar concentration of μM 4-hydroxy tamoxifen (Figure 5b). A 2-fold resistance to adriamycin remained with the highest concentration of 4-hydroxy tamoxifen, compared to the $IC_{50}$ of adriamycin alone (0.23 μM) in the CHO-K1 cell line.

*Effect of tamoxifen on intracellular accumulation of adriamycin*

To evaluate whether the potentiation of adriamycin cytotoxicity by tamoxifen could be attributed to increased accumulation of adriamycin, both cell lines were incubated with $^{14}C$-adriamycin for various time periods and levels of intracellular adriamycin measured.

Over a 120 min incubation period, there was a 1.5–2-fold lower amount of adriamycin in the CHO-Adr$^r$ cell line compared to the parental cell line (Figure 6a versus b, $P < 0.05$). The addition of 10 μM tamoxifen caused very small increases in drug levels in both cell lines (not significant).

*Effect of AAG on potentiation of adriamycin cytotoxicity by tamoxifen and 4-hydroxy tamoxifen*

Since tamoxifen and 4-hydroxy tamoxifen potentiated adriamycin cytotoxicity in CHO-K1 and CHO-Adr$^r$ cell lines, we

**Figure 5** **a**, Sensitivity of CHO-K1 cells to adriamycin in the absence (●—●) and presence of 5 μM (○—○), and 10 μM (□—□) 4-OH tamoxifen. Each point represents the mean ±s.e. of at least three experiments. **b**, Sensitivity of CHO-Adr$^r$ cells to adriamycin in the absence (▲—▲) and presence of 5 μM (△—△), 10 μM (■—■) 4-OH tamoxifen. Each point represents the mean±s.e. of at least three experiments.

assessed the effect of the addition of AAG in a concentration range present in cancer patients (Paxton, 1983). Both cell lines were accordingly exposed to equitoxic concentrations of adriamycin, in that concentration which reduced cell viability to 80–90% of control. Therefore, CHO-K1 cells in the presence of an increasing concentration range of AAG (0–2 mg ml$^{-1}$) were exposed to 0.05 μM and CHO-Adr$^r$ cells to 1 μM adriamycin in the absence or presence of 10 μM tamoxifen or 4-hydroxy tamoxifen.

In CHO-K1 cells (Figure 7a), adriamycin at 0.05 μM decreased cell viability to 86% of control and addition to 10 μM tamoxifen decreased it further to 37% of control. In CHO-Adr$^r$ cells, the cell viability was 82% of control in the presence of 1 μM adriamycin and was decreased to 44% of control with the addition of 10 μM tamoxifen. The addition of AAG (0–2 mg ml$^{-1}$) resulted in a gradual increase in cell viability, and finally, at 2 mg ml$^{-1}$ AAG, cell viability was similar to that of cells exposed to adriamycin alone.

Similarly, when 10 μM 4-hydroxy tamoxifen was added to adriamycin, cell viability was decreased to 35% of control in CHO-K1 cells and 35% of control in CHO-Adr$^r$ cells (Figure 7b). Addition of AAG increased cell survival and at the highest AAG concentration (2 mg ml$^{-1}$), there was no significant difference in the cell viability of cells treated with adriamycin alone versus cells treated with adriamycin, 4-hydroxy tamoxifen and AAG. AAG did not independently affect cell growth or adriamycin cytotoxicity (results not shown). However, at concentrations of AAG $\leqslant$ 1 mg ml$^{-1}$ potentiation was still detectable.

### Discussion

We have demonstrated a cytotoxic effect of tamoxifen and 4-hydroxy tamoxifen in the sensitive and resistant cell line. The CHO-Adr cell line is 28–30-fold resistant to adriamycin, but both cell lines demonstrate equal sensitivity to tamoxifen and 4-hydroxy tamoxifen, which suggests that different cytotoxic targets exist. 4-hydroxy tamoxifen has a 100-fold higher anti-oestrogen activity than tamoxifen, but its cytotoxicity is similar to tamoxifen. Therefore, we have concluded that the potentiating effect is independent of the oestrogen receptor status (no oestrogen receptors detectable by ligand binding were present in the two cell lines; results not shown). Since the cytotoxicity of 4-hydroxy tamoxifen was relatively unaltered by AAG compared to tamoxifen (Figure 1 and 2), it could be suggested that elevated AAG concentrations *in vivo* would cause less change in 4-hydroxy tamoxifen-mediated growth inhibition compared to tamoxifen. However, this effect was only demonstrated at toxic tamoxifen or 4-OH tamoxifen concentrations of greater than 10 μM, which are higher than those used for resistance modification.

We have also demonstrated in this study that the sensitivity to adriamycin in a MDR mutant (CHO-Adr$^r$) can be increased by tamoxifen or its metabolite 4-hydroxy tamoxifen at concentrations which do not inhibit cell growth on their own. The degree of potentiation was greater in the CHO-Adr$^r$ cell line than in the parent cell line, which is compatible with low degrees of *mdr* expression in the CHO-K1 cell line. This is similar to results with other modulators of MDR. The exact mechanism of modulation of adriamycin cytotoxicity is, however, unclear.

Reddel *et al.* (1985) have demonstrated that the growth inhibitor effect of tamoxifen is oestrogen-irreversible at high concentrations and they suggest the possibility of oestrogen-noncompatible, anti-oestrogen-specific binding sites (AEBS) (Sutherland *et al.*, 1980; Miller & Katzenellenbogen, 1983).

Ramu *et al.* (1984) have demonstrated reversal of MDR by triparonol analogues such as tamoxifen, clomiphene, nafoxidine (but not 4-hydroxy tamoxifen). They suggest that the

**Figure 6** **a**, Time course of uptake of 1 μM adriamycin in CHO-K1 cells in the absence (●—●) and presence (○—○) of 10 μM tamoxifen. Each point represents the mean±s.e. of at least three experiments. **b**, Time course of uptake 1 μM adriamycin in CHO-Adr$^r$ cells in the absence (▲—▲) and present (△—△) of 10 μM tamoxifen. Each point represents the mean±s.e. of at least three experiments.

**Figure 7** Sensitivity of CHO-K1 (●—●) and CHO-Adr (▲—▲) cell lines to 0.05 μM and 1.0 μM adriamycin respectively. Effect of increasing concentrations of AAG (0–2 mg ml$^{-1}$) in the presence of 10 μM tamoxifen (**a**) and 10 μM 4-OH tamoxifen in CHO-K1 (●—●) and CHO-Adr (▲—▲) cell lines. Each point represents the mean ± s.e. of at least three experiments.

increased membrane rigidity reported in MDR cell membranes was decreased by the triparonol analogues, which accounted for faster diffusion of adriamycin and enhancement of its cytotoxicity. Foster *et al.* (1988) have reported modulation of drug resistance in a MDR, MCF-7 breast cancer cell line with 10 μM tamoxifen or perhexilene maleate. Since the addition of 50 nM oestradiol did not attenuate the effects of tamoxifen, they have suggested that reversal of MDR by tamoxifen is not oestrogen-dependent. However, there was no increase in $^{14}$C-adriamycin accumulation, raising the possibility that tamoxifen modulates MDR by mechanisms other than increasing intracellular accumulation of the anticancer drugs to which the cell line is resistant. Kessel (1986) has studied the relationship between membrane transport systems involved with adriamycin, calcium antagonists (verapamil and nitrendipine) and anti-oestrogens (tamoxifen) in their circumvention of multidrug resistance. He concluded that no common exodus system can explain the effects of calcium antagonists and anti-oestrogens, both modulators of MDR. Thus these drugs modulate MDR by different membrane interactions. Yang *et al.* (1989) have demonstrated progesterone binds to P-glycoprotein, enhances drug accumulation and sensitivity of MDR cells to vinblastine. Their study also revealed that α and β-oestradiol do not bind to P-glycoprotein. Tamoxifen possibly does not bind to P-glycoprotein but reverse MDR by a P-glycoprotein independent mechanism. We have shown a 15-fold reversal in adriamycin cytotoxicity by 10 μM tamoxifen in CHO-Adr$^r$ cells (Figure 4b) but no significant increase in adriamycin accumulation with 10 μM tamoxifen (Figure 6b), which suggests that increased drug uptake is probably not one of the mechanism(s) by which tamoxifen reverses MDR.

Protein kinase C (PKC) is a high affinity phorbol ester receptor. Phorbol esters and other tumour promoters function by acting as diglyceride substitutes and active PKC *in vitro* and *in vivo*. PKC is believed to transduce a variety of growth promoting signals and may have an important role in tumour promotion. The importance of PKC is regulation of cell growth suggests that PKC inhibitors could prove to be effective anti-proliferative agents. O'Brian *et al.* (1988) have reported (a) inhibition of rat PKC activity *in vitro* by tamoxifen and its principal metabolites 4-hydroxy tamoxifen and desmethyl tamoxifen, mediated by the compounds binding to the catalytic domain of the enzyme and (b) the inhibitory potencies against PKC activity correlate with the oestrogen irreversible cytotoxic effects shown in the MCF-7 cell line. Horgan *et al.* (1986) have shown inhibition of PKC activity *in vivo* by tamoxifen. These results, therefore, suggest that inhibition of PKC may play an important role in the anti-tumour effect and modulation of MDR by tamoxifen and 4-hydroxy tamoxifen.

Another more likely target is calmodulin since the $IC_{50}$ of tamoxifen is only 2 μM for this enzyme (Lam, 1984) compared with 25 μM for $IC_{50}$ of 4-hydroxy tamoxifen on PKC (O'Brian *et al.*, 1988).

AAG, which is normally absent from cell culture medium, has been shown to reverse the effect of verapamil-induced potentiation of adriamycin cytotoxicity (Chatterjee *et al.*, in preparation). Verapamil binding to AAG has been shown by Gillis *et al.* (1985). Since tamoxifen was reported to be 99% bound to albumin (Adam, 1981) and binding to AAG had not been suggested, we wanted to assess whether AAG, when present, could alter the reversal of adriamycin resistance by tamoxifen or 4-hydroxy tamoxifen. Our results have shown that AAG present at concentrations found at the higher range in cancer patients (0.8–2.0 mg ml$^{-1}$) can attenuate the reversal of multidrug resistance. However, AAG at levels found in the normal population and at the lower end of the cancer population allowed for enhancement of adriamycin cytotoxicity by the modulators used. Lien *et al.* (1989) recently reported that tamoxifen is bound mainly to albumin, but only assessed binding of approximately 40 nM tamoxifen. In our study, 250-fold higher levels were used and it is clear that at these levels AAG markedly modifies the effects of tamoxifen and tamoxifen binds to AAG in the presence of albumin.

There was a residual 3-fold resistance of the CHO-Adr$^r$ mutant to adriamycin in the presence of tamoxifen. Recent studies have shown it is possible to increase the dose of adriamycin more than 2-fold, provided marrow is supported by haemopoetic growth factors (Bronchud *et al.*, 1989). Thus, a combined approach may be able to reverse resistance clinically. Although normal tissues may also be sensitised, the relatively greater effects of reversal agents on resistant cells with high levels of expression may still enhance the therapeutic/toxic ratio of anthracycline.

Possible clinical implications of this study are that tamoxifen and 4-hydroxy tamoxifen could prove effective cytotoxic agents as well as modulators of multidrug resistance; the limiting factor to their effectiveness could be high levels of AAG. It is conceivable that the free fraction of tamoxifen diffuses into tissues over several days to weeks and accumulates there, to exert its anti-oestrogen effect. Although 4-hydroxy tamoxifen is a minor metabolite of tamoxifen, it is much more potent (Jordan *et al.*, 1977). It has a lower degree of AAG binding, as assessed from the lack of protection by AAG against 4-hydroxy tamoxifen toxicity. Thus *in vivo* it may be a major component of the biological anti-oestrogen effect. Clinical trials with the addition of tamoxifen in a chemotherapy regimen could increase the therapeutic index of the anticancer agents, in tumours without oestrogen receptors. It would be appropriate to select patients with low AAG levels for such studies.

We thank the North of England Cancer Research Campaign for support.

### References

ADAM, H.K. (1981). A review of the pharmacokinetics and metabolism of tamoxifen. In *Non Steroidal Anti-oestrogens. Molecular Pharmacology and Antitumour Activity*, Sutherland, R.L. (ed.) p. 59. Academic Press: London.

BENSON, A.B. III, TRUMP, D.L., KOELLER, J.M. & 5 others (1985). Phase I study of vinblastine and verapamil given by concurrent I.V. infusion. *Cancer Treat. Rep.*, **69**, 795.

BIEDLER, J.L. & RIEHM, H. (1970). Cellular resistance to actinomycin D in CHO cells in vitro: cross resistance, radioautographic and cytogenetic studies. *Cancer Res.*, **30**, 1174.

BRONCHUD, M.H., HOWELL, A., CROWTHER, D., HOPWOOD, P., SOUZA, L. & DEXTER, T.M. (1989). The use of granulocyte colony-stimulating factor to increase the intensity of treatment with doxorubicin in patients with advanced breast and ovarian cancer. *Br. J. Cancer*, **60**, 121.

CARMICHAEL, J., DEGRAFF, W.G., GAZDAR, A.F., MINNA, J.D. & MITCHELL, J.B. (1987). Evaluation of a tetrazolium based semiautomated colorimetric assay: assessment of chemosensitivity testing. *Cancer Res.*, **47**, 936.

CHAUFFERT, B., MARTIN, M., HAMMAN, A., MICHEL, M.F. & MARTIN, F. (1986). Amiodarone enhancement of doxorubicin and 4'-deoxydoxorubicin cytotoxicity to rat colon cancer cells in vitro and in vivo. *Cancer Res.*, **46**, 825.

DANO, K. (1973). Active outward transport of daunorubicin in resistant Ehrlich ascites tumour cells. *Biochim. Biophys. Acta*, **323**, 466.

FOJO, A., AKIYAMA, S.I., GOTTESMAN, M.M. & PASTAN, I. (1985). Reduced drug accumulation in multiply drug resistant human KB carcinoma cell lines. *Cancer Res.*, **45**, 3002.

FOSTER, B.J., GROTZINGER, K.R., MCKOY, W.M., RUBINSTEIN, L.V. & HAMILTON, T.C. (1988). Modulation of induced resistance to Adriamycin in two human breast cancer cell lines with tamoxifen or perhexilene maleate. *Cancer Chemother. Pharmacol.*, **22**, 147.

GILLIS, A.N., YEE, Y. & KATES, R.E. (1985). Binding of antiarrhythmic drugs to purified human alpha 1 acid glycoprotein. *Biochem. Pharm.*, **34**, 4279.

GROS, P., CROOP, J., RONINSON, I., VARSHAVSKY, A. & HOUSMAN, D.E. (1986). Isolation and characterization of DNA sequences amplified in multidrug resistant hamster cells. *Proc. Natl Acad. Sci. USA*, **83**, 337.

HORGAN, K., COOKE, E., HALLETT, M.B. & MANSEL, R.E. (1986). Inhibition of protein kinase C mediated signal transduction by tamoxifen. *Biochem. Pharm.*, **35**, 4463.

INABA, M., KOBAYASHI, H., SAKURAI, Y. & JOHNSON, R.K. (1979). Active efflux of daunorubicin and Adriamycin in sensitive and resistant sublines of P388 leukaemia. *Cancer Res.*, **39**, 2200.

JORDAN, V.C., COLLINS, M.M., ROWSBY, L. & PRESTWICH, G. (1977). A monohydroxylated metabolite of tamoxifen with potent antiestrogenic activity. *J. Endocrinol.*, **75**, 305.

KARTNER, N., EVERNDEN-PORELLE, D., BRADLEY, G. & LING, V. (1985). Detection of P-glycoprotein in multidrug resistant cell lines by monoclonal antibodies. *Nature*, **316**, 820.

KARTNER, N., RIORDAN, J.R. & LING, V. (1983). Cell surface P-glycoprotein associated with multidrug resistance in mammalian cell lines. *Science*, **221**, 1285.

KESSEL, D. (1986). Interactions among membrane transport systems: anthracyclines, calcium antagonists and anti-oestrogens. *Biochem. Pharm.*, **35**, 2825.

LAM, H.-Y.P. (1984). Tamoxifen is a calmodulin antagonist in the activation of cAMP phosphodiesterase. *BBRC*, **118**, 27.

LIEN, E.A., SOLHEIM, E., LEA, O.A., LUNDGREN, S., KVINNSLAND, S. & UELAND, P.M. (1989). Distribution of 4-Hydroxy-N-desmethyltamoxifen and other tamoxifen metabolites in human biological fluids during tamoxifen treatment. *Cancer Res.*, **49**, 2175.

MILLER, M.A. & KATZENELLENBOGEN, B.S. (1983). Characterization and quantitation of anti-oestrogen binding sites in estrogen receptor-positive and -negative human breast cancer cell lines. *Cancer Res.*, **43**, 3094.

O'BRIAN, C.A., WARD, N.E. & ANDERSON, B.W. (1988). Role of specific interactions between protein kinase C and triphenylethylenes in inhibition of the enzyme. *J. Natl Cancer Inst.*, **80**, 1628.

PATTERSON, J.S. (1981). Clinical aspects and developments of anti-oestrogen therapy: a review of the endocrine effects of tamoxifen in animal and man. *J. Endocrinol.*, **89**, 67.

PAXTON, J.W. (1983). Alpha 1 acid glycoprotein and binding of basic drugs. *Meth. Find. Exp. Clin. Pharm.*, **5**, 635.

RAMU, A., FUKS, Z., GATT, S. & GLAUBIGER, D. (1984*a*). Reversal of acquired resistance to doxorubicin in P388 murine leukaemia cells by perhexilene maleate. *Cancer Res.*, **44**, 144.

RAMU, A., GLAUBIGER, D. & FUKS, Z. (1984*b*). Reversal of acquired resistance to doxorubicin in P388 murine leukaemia cells by tamoxifen and triparanol analogues. *Cancer Res.*, **44**, 4392.

REDDEL, R.R., MURPHY, L.C., HALL, R.E. & SUTHERLAND, R.L. (1985). Differential sensitivity of human breast cancer cell lines to the growth inhibitory effects of tamoxifen. *Cancer Res.*, **45**, 1525.

ROBERTSON, D.W., KATZENELLENBOGEN, J.A., LONG, D.J., RORKE, E.A. & KATZENELLENBOGEN, B.S. (1982). Tamoxifen antiestrogens. A comparison of the activity, pharmacokinetics and metabolic activation of the cis and trans isomers of tamoxifen. *J. Steroid Biochem.*, **16**, 1.

ROGAN, A.M., HAMILTON, T.C., YOUNG, R.C., KLECKER, R.W. Jr & OZOLS, R.F. (1984). Reversal of Adriamycin resistance by verapamil in human ovarian cancer. *Science*, **224**, 994.

SAFA, A.R., GLOVER, C.J., SEWELL, J.L., MEYERS, M.B., BIEDLER, J.L. & FELSTED, R.L. (1987). Identification of the multidrug resistance related membrane glycoprotein as an acceptor for calcium channel blockers. *J. Biol. Chem.*, **262**, 7884.

SUTHERLAND, R.L., MURPHY, L.C., FOO, M.S., GREEN, M.D. & WHYBOURNE, A.M. (1980). High-affinity anti-oestrogen binding site distinct from the oestrogen receptor. *Nature*, **288**, 273.

TSURUO, T., IIDA, H., TSUKAGOSHI, S. & SAKURAI, Y. (1982). Increased accumulation of vincristine and Adriamycin in drug resistant P388 tumour cells following incubation with calcium antagonists and calmodulin inhibitors. *Cancer Res.*, **42**, 4730.

TSURUO, T., IIDA, H., TSUKAGOSHI, S. & SAKURAI, Y. (1981). Overcoming the vincristine resistance of P388 leukaemia *in vivo* and *in vitro* enhanced cytotoxicity of vincristine and vinblastine by verapamil. *Cancer Res.*, **41**, 1976.

YANG, C.H., DEPINHO, S.G., GREENBERGER, L.M., ARCECI, R.J. & HORWITZ, S.B. (1989). Progesterone interacts with P-glycoprotein in multidrug-resistant cells and in the endometrium of gravid uterus. *J. Biol. Chem.*, **264**, 782.


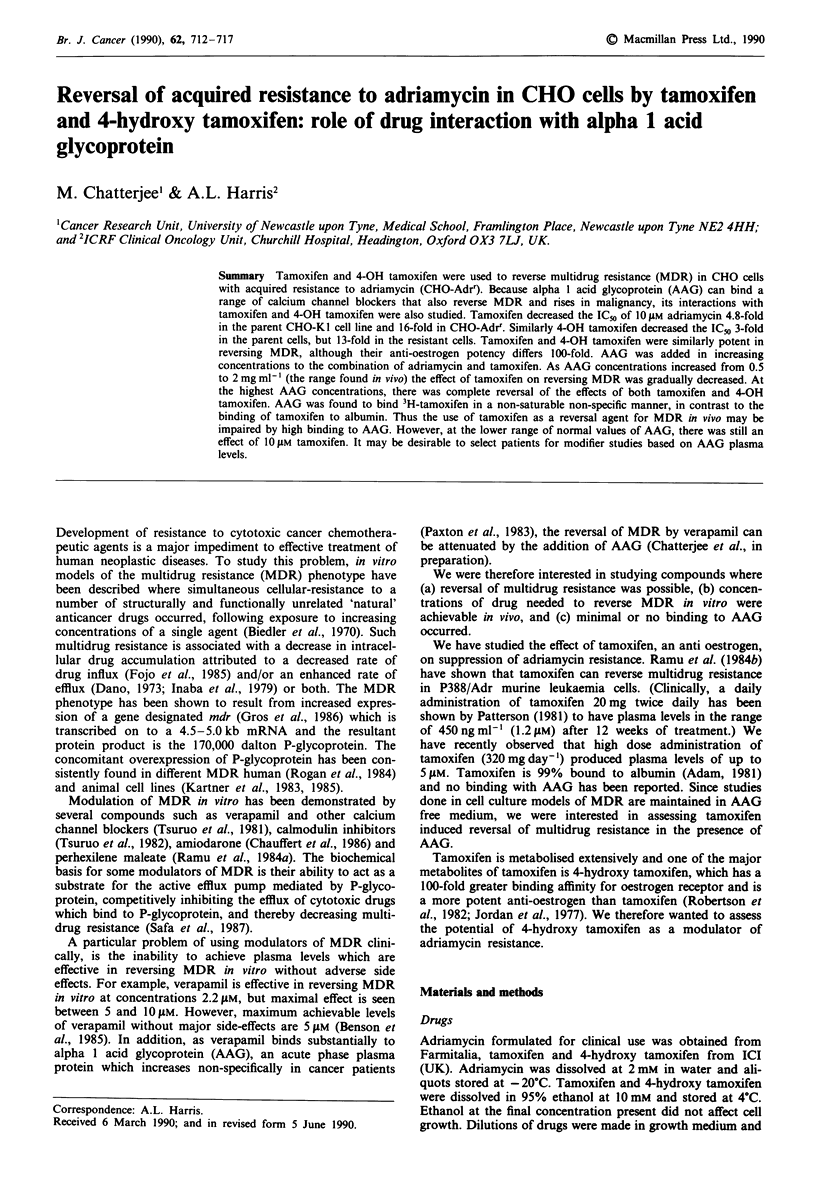

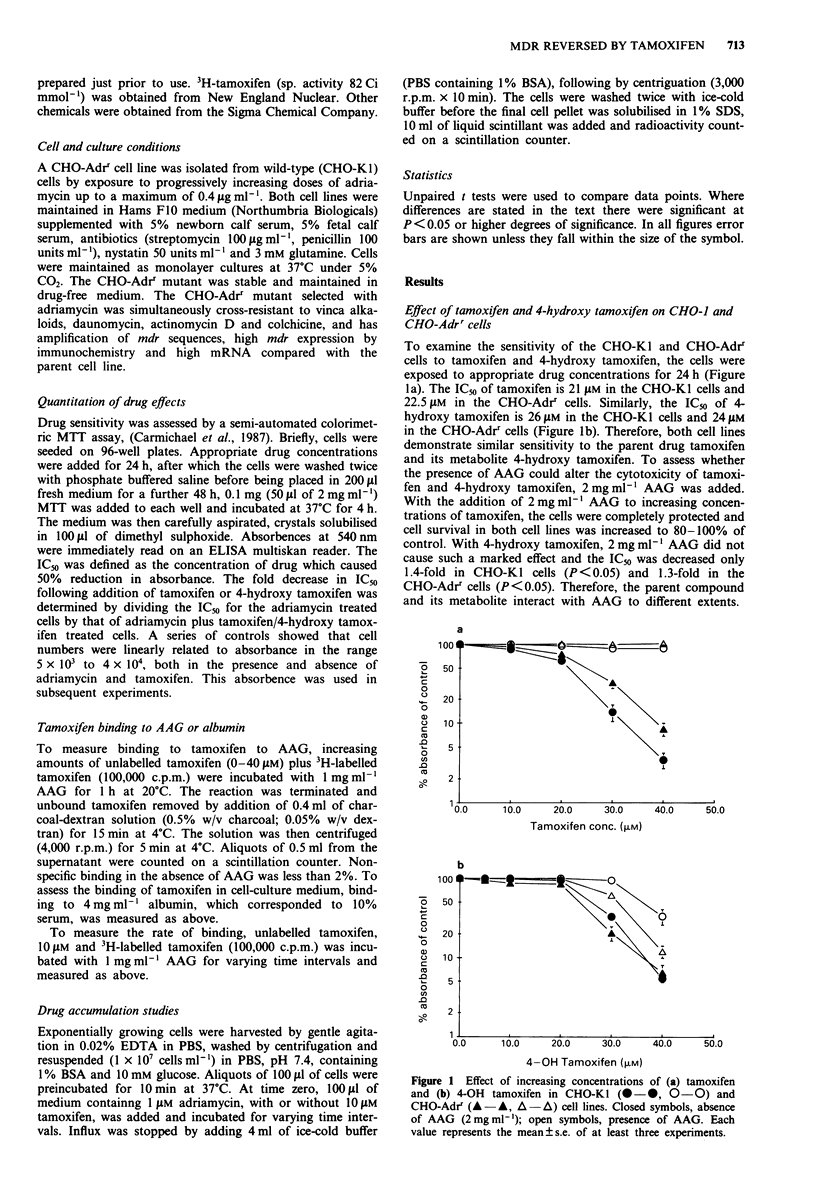

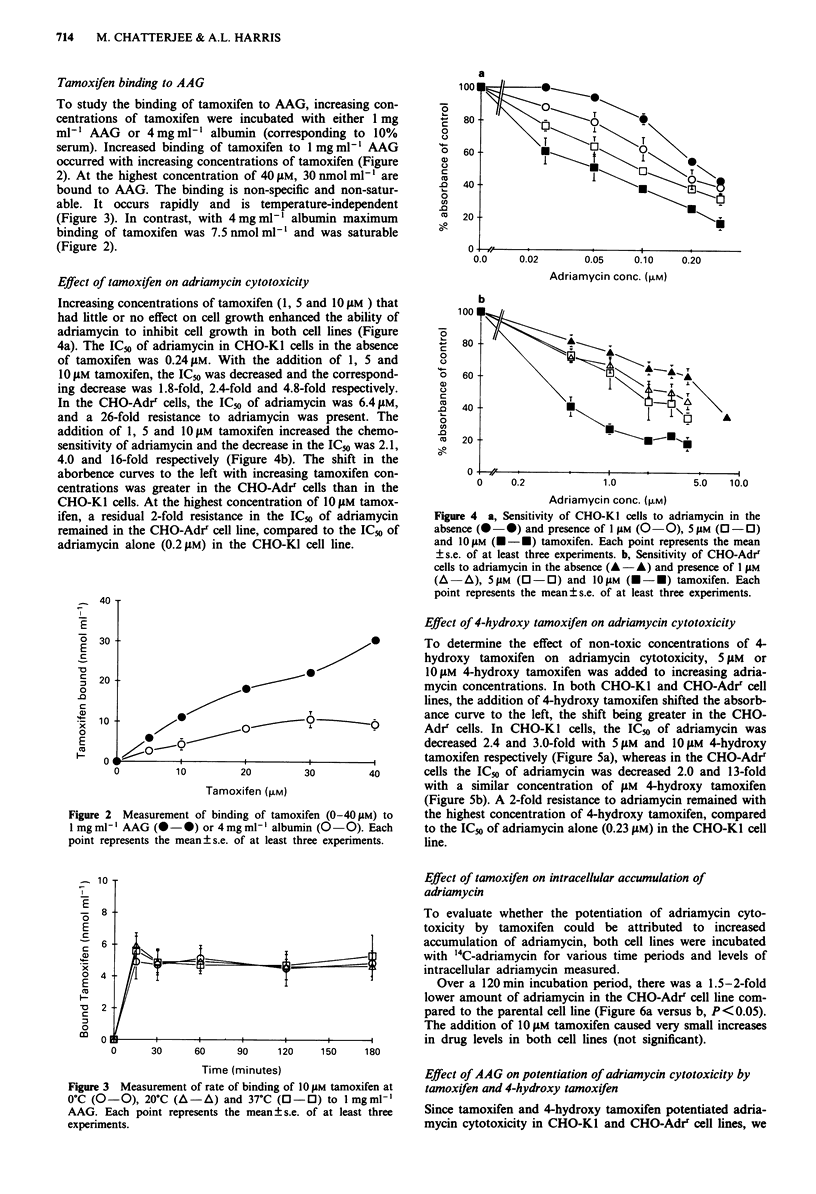

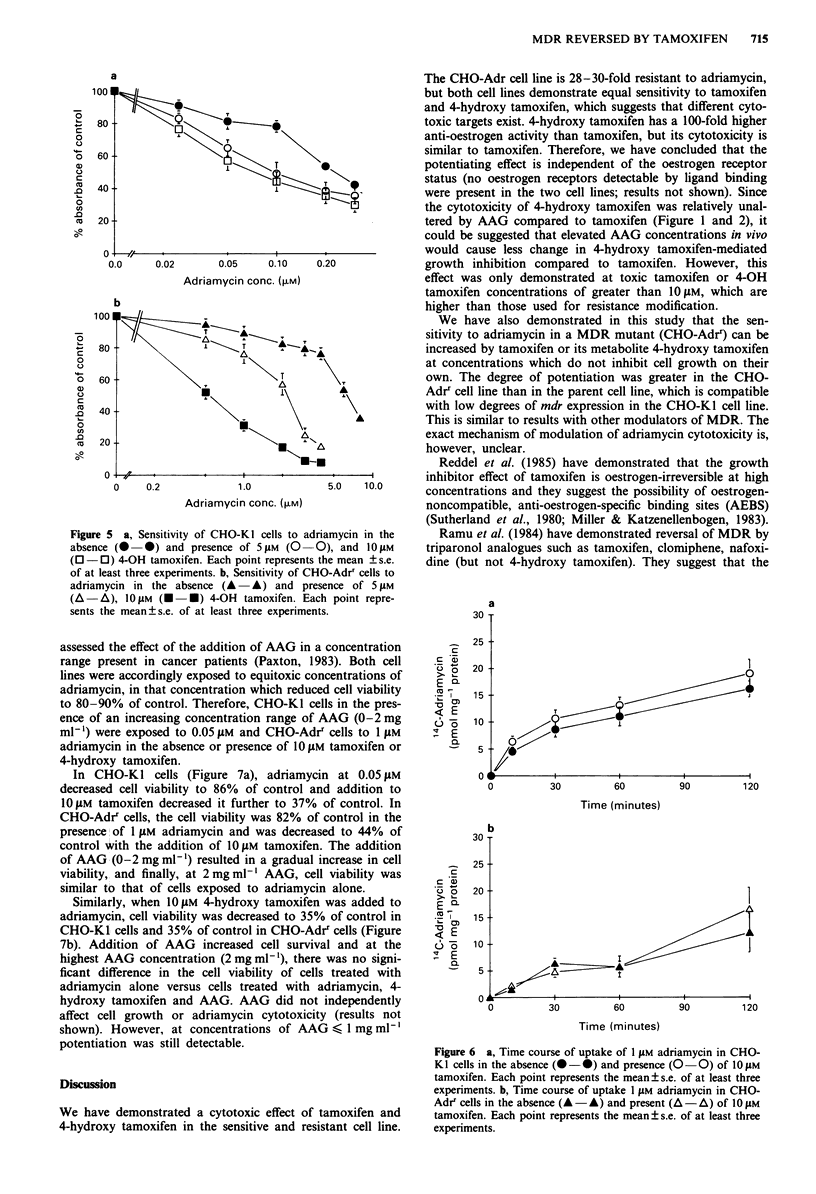

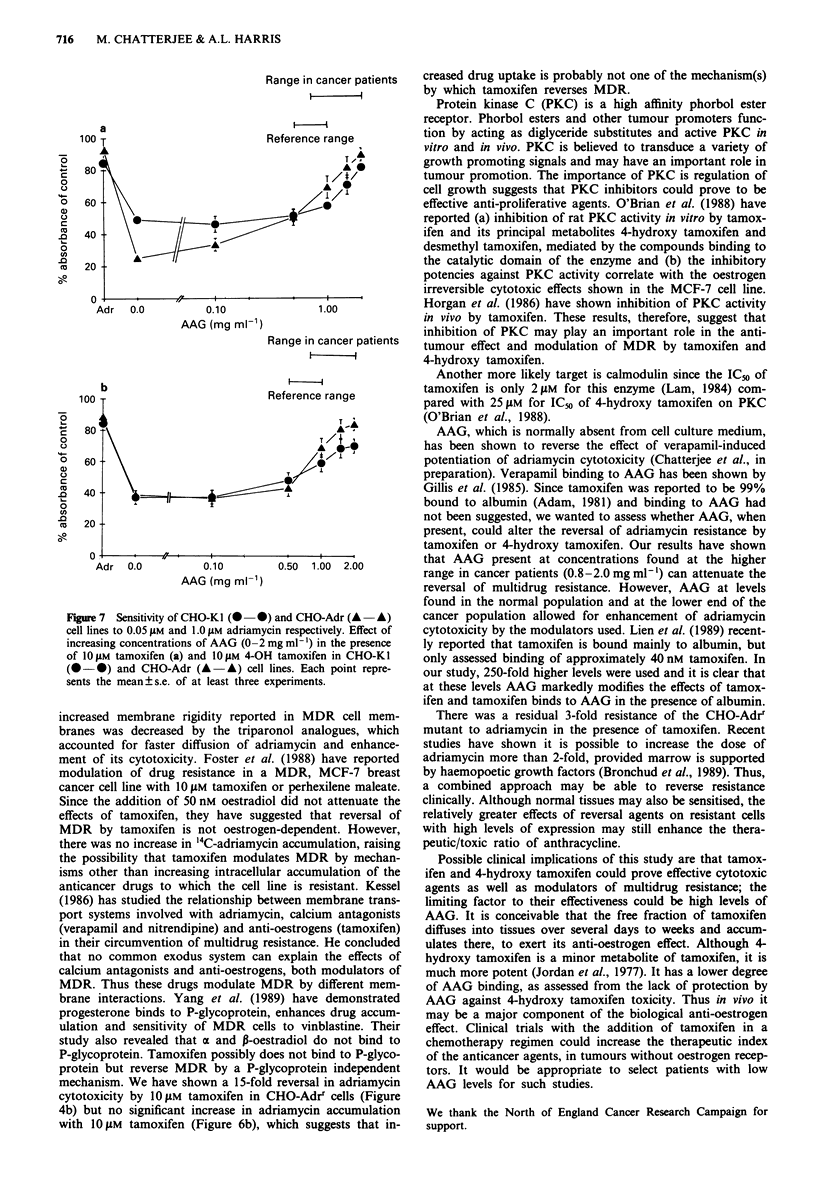

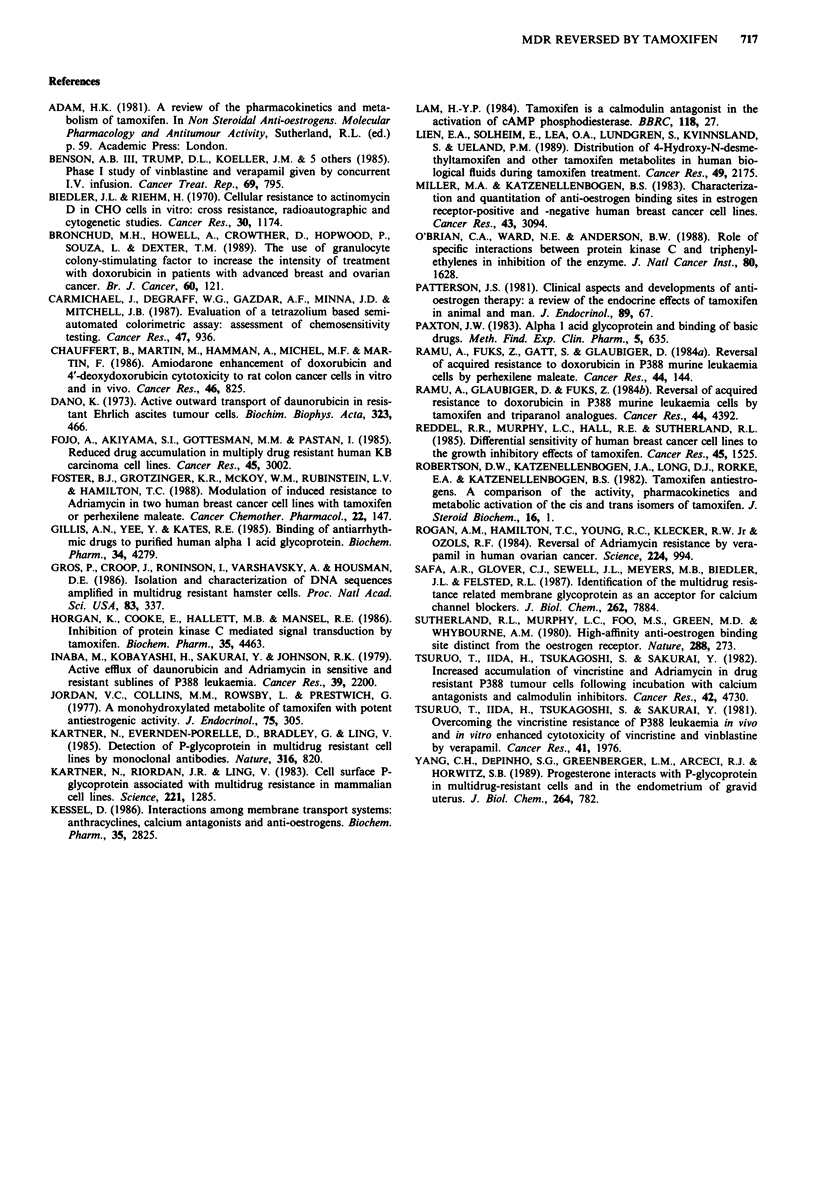


## References

[OCR_00985] Benson A. B., Trump D. L., Koeller J. M., Egorin M. I., Olman E. A., Witte R. S., Davis T. E., Tormey D. C. (1985). Phase I study of vinblastine and verapamil given by concurrent iv infusion.. Cancer Treat Rep.

[OCR_00990] Biedler J. L., Riehm H. (1970). Cellular resistance to actinomycin D in Chinese hamster cells in vitro: cross-resistance, radioautographic, and cytogenetic studies.. Cancer Res.

[OCR_00995] Bronchud M. H., Howell A., Crowther D., Hopwood P., Souza L., Dexter T. M. (1989). The use of granulocyte colony-stimulating factor to increase the intensity of treatment with doxorubicin in patients with advanced breast and ovarian cancer.. Br J Cancer.

[OCR_01002] Carmichael J., DeGraff W. G., Gazdar A. F., Minna J. D., Mitchell J. B. (1987). Evaluation of a tetrazolium-based semiautomated colorimetric assay: assessment of chemosensitivity testing.. Cancer Res.

[OCR_01010] Chauffert B., Martin M., Hammann A., Michel M. F., Martin F. (1986). Amiodarone-induced enhancement of doxorubicin and 4'-deoxydoxorubicin cytotoxicity to rat colon cancer cells in vitro and in vivo.. Cancer Res.

[OCR_01014] Dano K. (1973). Active outward transport of daunomycin in resistant Ehrlich ascites tumor cells.. Biochim Biophys Acta.

[OCR_01019] Fojo A., Akiyama S., Gottesman M. M., Pastan I. (1985). Reduced drug accumulation in multiply drug-resistant human KB carcinoma cell lines.. Cancer Res.

[OCR_01024] Foster B. J., Grotzinger K. R., McKoy W. M., Rubinstein L. V., Hamilton T. C. (1988). Modulation of induced resistance to adriamycin in two human breast cancer cell lines with tamoxifen or perhexiline maleate.. Cancer Chemother Pharmacol.

[OCR_01029] Gillis A. M., Yee Y. G., Kates R. E. (1985). Binding of antiarrhythmic drugs to purified human alpha 1-acid glycoprotein.. Biochem Pharmacol.

[OCR_01034] Gros P., Croop J., Roninson I., Varshavsky A., Housman D. E. (1986). Isolation and characterization of DNA sequences amplified in multidrug-resistant hamster cells.. Proc Natl Acad Sci U S A.

[OCR_01040] Horgan K., Cooke E., Hallett M. B., Mansel R. E. (1986). Inhibition of protein kinase C mediated signal transduction by tamoxifen. Importance for antitumour activity.. Biochem Pharmacol.

[OCR_01045] Inaba M., Kobayashi H., Sakurai Y., Johnson R. K. (1979). Active efflux of daunorubicin and adriamycin in sensitive and resistant sublines of P388 leukemia.. Cancer Res.

[OCR_01050] Jordan V. C., Collins M. M., Rowsby L., Prestwich G. (1977). A monohydroxylated metabolite of tamoxifen with potent antioestrogenic activity.. J Endocrinol.

[OCR_01055] Kartner N., Evernden-Porelle D., Bradley G., Ling V. Detection of P-glycoprotein in multidrug-resistant cell lines by monoclonal antibodies.. Nature.

[OCR_01060] Kartner N., Riordan J. R., Ling V. (1983). Cell surface P-glycoprotein associated with multidrug resistance in mammalian cell lines.. Science.

[OCR_01065] Kessel D. (1986). Interactions among membrane transport systems: anthracyclines, calcium antagonists and anti-estrogens.. Biochem Pharmacol.

[OCR_01070] Lam H. Y. (1984). Tamoxifen is a calmodulin antagonist in the activation of cAMP phosphodiesterase.. Biochem Biophys Res Commun.

[OCR_01074] Lien E. A., Solheim E., Lea O. A., Lundgren S., Kvinnsland S., Ueland P. M. (1989). Distribution of 4-hydroxy-N-desmethyltamoxifen and other tamoxifen metabolites in human biological fluids during tamoxifen treatment.. Cancer Res.

[OCR_01079] Miller M. A., Katzenellenbogen B. S. (1983). Characterization and quantitation of antiestrogen binding sites in estrogen receptor-positive and -negative human breast cancer cell lines.. Cancer Res.

[OCR_01085] O'Brian C. A., Ward N. E., Anderson B. W. (1988). Role of specific interactions between protein kinase C and triphenylethylenes in inhibition of the enzyme.. J Natl Cancer Inst.

[OCR_01096] Paxton J. W. (1983). Alpha 1 -acid glycoprotein and binding of basic drugs.. Methods Find Exp Clin Pharmacol.

[OCR_01100] Ramu A., Fuks Z., Gatt S., Glaubiger D. (1984). Reversal of acquired resistance to doxorubicin in P388 murine leukemia cells by perhexiline maleate.. Cancer Res.

[OCR_01105] Ramu A., Glaubiger D., Fuks Z. (1984). Reversal of acquired resistance to doxorubicin in P388 murine leukemia cells by tamoxifen and other triparanol analogues.. Cancer Res.

[OCR_01110] Reddel R. R., Murphy L. C., Hall R. E., Sutherland R. L. (1985). Differential sensitivity of human breast cancer cell lines to the growth-inhibitory effects of tamoxifen.. Cancer Res.

[OCR_01114] Robertson D. W., Katzenellenbogen J. A., Long D. J., Rorke E. A., Katzenellenbogen B. S. (1982). Tamoxifen antiestrogens. A comparison of the activity, pharmacokinetics, and metabolic activation of the cis and trans isomers of tamoxifen.. J Steroid Biochem.

[OCR_01121] Rogan A. M., Hamilton T. C., Young R. C., Klecker R. W., Ozols R. F. (1984). Reversal of adriamycin resistance by verapamil in human ovarian cancer.. Science.

[OCR_01126] Safa A. R., Glover C. J., Sewell J. L., Meyers M. B., Biedler J. L., Felsted R. L. (1987). Identification of the multidrug resistance-related membrane glycoprotein as an acceptor for calcium channel blockers.. J Biol Chem.

[OCR_01132] Sutherland R. L., Murphy L. C., San Foo M., Green M. D., Whybourne A. M., Krozowski Z. S. (1980). High-affinity anti-oestrogen binding site distinct from the oestrogen receptor.. Nature.

[OCR_01137] Tsuruo T., Iida H., Tsukagoshi S., Sakurai Y. (1982). Increased accumulation of vincristine and adriamycin in drug-resistant P388 tumor cells following incubation with calcium antagonists and calmodulin inhibitors.. Cancer Res.

[OCR_01149] Yang C. P., DePinho S. G., Greenberger L. M., Arceci R. J., Horwitz S. B. (1989). Progesterone interacts with P-glycoprotein in multidrug-resistant cells and in the endometrium of gravid uterus.. J Biol Chem.

